# Physical Activity and Systemic Lupus Erythematosus Among European Populations: A Two-Sample Mendelian Randomization Study

**DOI:** 10.3389/fgene.2021.784922

**Published:** 2022-02-08

**Authors:** Shuo Huang, Fengyuan Tian, Xiaoxuan Yang, Sijia Fang, Yongsheng Fan, Jie Bao

**Affiliations:** ^1^ The First School of Clinical Medicine, Zhejiang Chinese Medical University, Hangzhou, China; ^2^ School of Basic Medical Sciences, Zhejiang Chinese Medical University, Hangzhou, China

**Keywords:** physical activity, sedentary behavior, systemic lupus erythematosus, single nucleotide polymorphism, mendelian randomization

## Abstract

**Background:** The causal relationship between physical activity (PA) and systemic lupus erythematosus (SLE) remains uncertain. We aimed to assess the causal effect of PA on SLE by two-sample Mendelian randomization (MR) study.

**Methods:** Summary statistics of SLE were obtained from a genome-wide association study (GWAS) meta-analysis of European descent, including 4,036 cases and 6,959 controls. Genetic instruments for PA, including MVPA, VPA, SSOE, and average acceleration, were identified from a large-scale GWAS meta-analysis among 377,234 individuals of European ancestry from United Kingdom biobank and Atherosclerosis Risk in Communities (ARIC) study, and another GWAS with 91,105 European participants was employed for sedentary behavior. The two-sample MR study was conducted to estimate causal relationship between PA and SLE, with the inverse-variance weighted (IVW) method, simple- and weighted-median method. Moreover, MR-Egger regression, MR-PRESSO and leave-one-out analysis were performed to evaluate the potential pleiotropy effect.

**Results:** In the end, we totally selected 37 SNPs (15 SNPs for MVPA, 5 SNPs for VPA, 9 SNPs for SSOE, 5 SNPs for average acceleration and 3 SNPs for sedentary behavior). According to the IVW results, as the primary method, we found that genetically predicted PA was not causally associated with risk of SLE (MVPA: OR 0.44, 95% CI 0.09–2.10, *p* = 0.305; VPA: OR 0.20, 95% CI 0.00–18.97, *p* = 0.490; SSOE: OR 0.96, 95% CI 0.03–29.24, *p* = 0.983; average acceleration: OR 0.91, 95% CI 0.79–1.05, *p* = 0.190; sedentary behavior: OR 1.54, 95% CI 0.35–6.81, *p* = 0.572). MR-Egger, MR-PRESSO, and leave-one-out analysis did not indicate horizontal pleiotropy.

**Conclusions:** Our MR study suggested that genetically predicted PA was not causally associated with SLE among the European populations.

## Introduction

Systemic lupus erythematosus (SLE) is a prototypical systemic autoimmune disease, clinically characterized by the involvement of multiple organs and the production of various autoantibodies (Guo et al., 2018). The prevalence of SLE has been estimated to be 30–50 per 100,000, which equates to ∼500,000 patients in Europe (Dorner and Furie, 2019). Although the survival rate of SLE was apparently enhanced with the continuous improvement of diagnosis and treatment, the all-cause mortality for SLE patients is still over twice greater than that for the general population (Lee et al., 2016).

Physical activity (PA) has been recognized as an essential component of health since antiquity. PA can reduce the risk of cardiovascular diseases (CVDs) and multiple metabolic diseases (Reddigan et al., 2011). Particularly, it has been demonstrated to regulate the immune system, increasing T-regulatory (Treg) cells and decreasing immunoglobulin secretion (Tharp and Barnes, 1990; Weinhold et al., 2016). Previous studies supported that PA was associated with a risk reduction in incidence of several systemic inflammatory diseases, including rheumatoid arthritis (RA), multiple sclerosis (MS), SLE, as well as others (Sharif et al., 2018). Nevertheless, sedentary behavior is one of the most serious health problems worldwide and an important risk factor in numerous autoimmune rheumatic diseases (Kohl et al., 2012; Pinto et al., 2017). Depending on modality and dramatic extent, self-reported levels of PA were grouped into vigorous PA (VPA), moderate-to-vigorous PA (MVPA) and strenuous sports or other exercises (SSOE) (Klimentidis et al., 2018). To avoid information bias, the levels of PA could be objectively reflected by overall average acceleration which was measured by wearing a wrist-worn accelerometer (Doherty et al., 2017).

Significantly, there exist several discrepancies between different epidemic studies. For instance, 60% of patients with SLE did not meet levels of PA for health, which was advocated by the World Health Organization (WHO) (Margiotta et al., 2018), and moderate PA was helpful to recover from their conditions (Eriksson et al., 2012; Wu et al., 2017). However, several studies failed to indicate that PA was relevant to reduced disease activity of SLE (Abrahao et al., 2016; Bostrom et al., 2016; Fangtham et al., 2019). These different results could be possibly driven by confounders and inverse causality (Smith and Ebrahim, 2004). Therefore, it is necessary to explore the causal relationship between PA and SLE.

Mendelian randomization (MR) is increasingly used as an approach to assess causal association in epidemiology by using genetic variants as instrumental variables (IVs) (Smith and Ebrahim, 2003; Davey Smith and Hemani, 2014). As genotypes precede the diseases process and are largely independent of postnatal environmental factors or lifestyle, MR can avoid several factors interference, such as confounding factors, reverse causation, selection biases, etc. (Smith and Ebrahim, 2004). Compared with one-sample MR, two-sample MR evaluates the causality between exposure and outcome among completely independent populations (Julian et al., 2021).

In this study, we aimed to estimate the causal effect of five types of PA (MVPA, VPA, SSOE, average acceleration, and sedentary behavior) with risk of SLE by two-sample MR.

## Materials and methods

**FIGURE 1:**
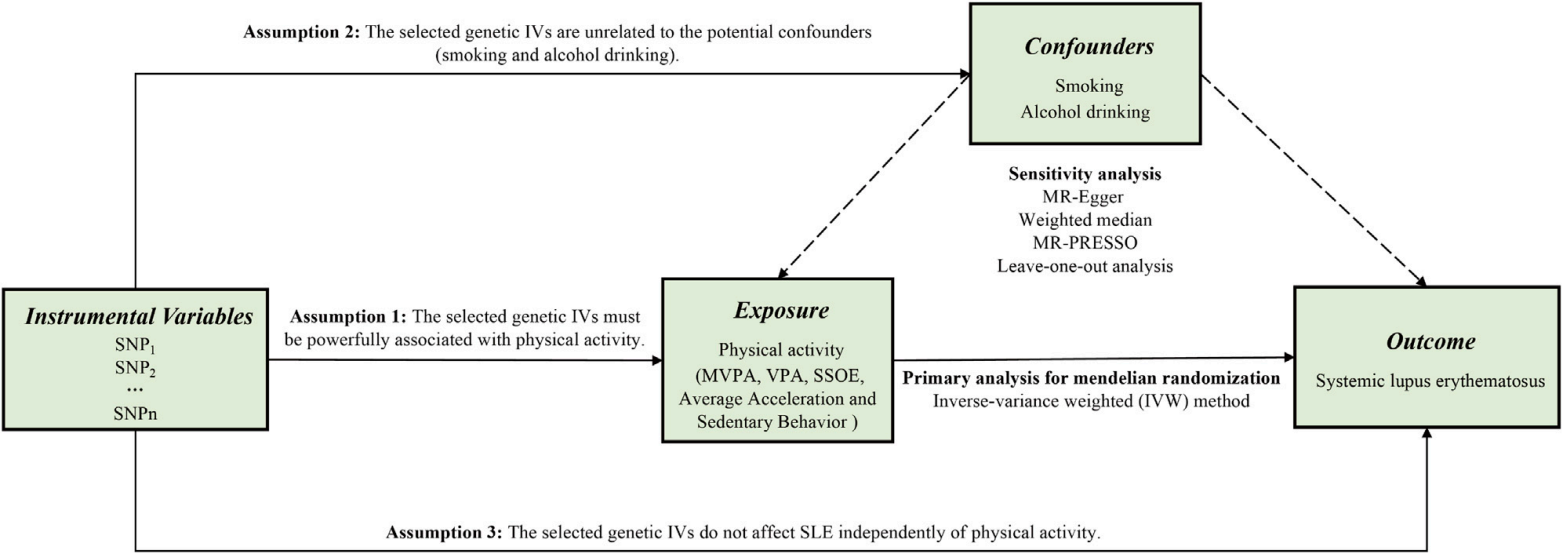
An overview of this Mendelian randomization (MR) study design. Abbreviations: SNP, single nucleotide polymorphism; MVPA, Moderate-to-vigorous physical activity; VPA, vigorous physical activity; SSOE, strenuous sports or other exercises; IVs, instrumental variables; SLE, systemic lupus erythematosus; MR-Egger, Mendelian randomization-Egger; MR-PRESSO, Mendelian randomization pleiotropy residual sum and outlier test.

Supposing the causal estimate of MR studies is persuasive, three pivotal assumptions must be met: 1) The selected genetic IVs must be powerfully associated with exposure (Lawlor et al., 2008). 2) The selected genetic IVs do not affect outcome independently of exposure (i.e., horizontal pleiotropy is nonexistent) (Bowden et al., 2016). 3) The selected genetic IVs are unrelated to the potential confounders. Figure 1 shows an overview of the current study design. Ethical approval and consent to participants were not necessary as the study was based on openly available databases and published studies.

### Data sources

In terms of the exposure, the genome-wide association study (GWAS) meta-analysis with 377,234 participants of European ancestry was applied to select IVs for MVPA, VPA, SSOE, and average acceleration (Klimentidis et al., 2018). These participants came from the United Kingdom Biobank study and the Atherosclerosis Risk in Communities (ARIC) study. The majority of United Kingdom Biobank participants were genotyped with the Affymetrix United Kingdom Biobank Axiom Array, 10% with the Affymetrix United Kingdom BiLEVE Axiom Array. Besides, in ARIC, participants were genotyped with the Affymetrix Genome-Wide Human SNP Array 6.0. Additionally, another GWAS with 91,105 European participants was employed for sedentary behavior (Doherty et al., 2018).

To avoid participant overlap, genetic data on SLE were obtained from a previous GWAS meta-analysis, not United Kingdom Biobank participants, including 10,995 subjects with European ancestry (4,036 cases and 6,959 controls) in total, covering 644,674 single nucleotide polymorphisms (SNPs) (Bentham et al., 2015). They genotyped 4,036 SLE cases and 1,260 controls by the Illumina HumanOmni1-Quad BeadChip. Besides, they also used data for 5,699 previously genotyped controls taken from the University of Michigan Health and Retirement Study (HRS). These individuals were genotyped by the Illumina Human2.5M Beadchip. All cases met the standard American College of Rheumatology (ACR) classification criteria for diagnosis of SLE.

As for the confounders, genetic variants associated with smoking (cigarettes/d) and alcohol drinking (alcoholic drinks/w) were based on a large-scale available GWAS with 1,232,091 individuals of European ancestry (Liu et al., 2019). All participants were genotyped on genome-wide arrays. Supplementary Table S1 showed basic characteristics of relevant GWAS studies and data sources. The quality control and information of genetic variants and imputation methods of missing data were described in original manuscripts (Bentham et al., 2015; Doherty et al., 2017; Klimentidis et al., 2018; Liu et al., 2019).

### Selection of instrumental variables

**FIGURE 2:**
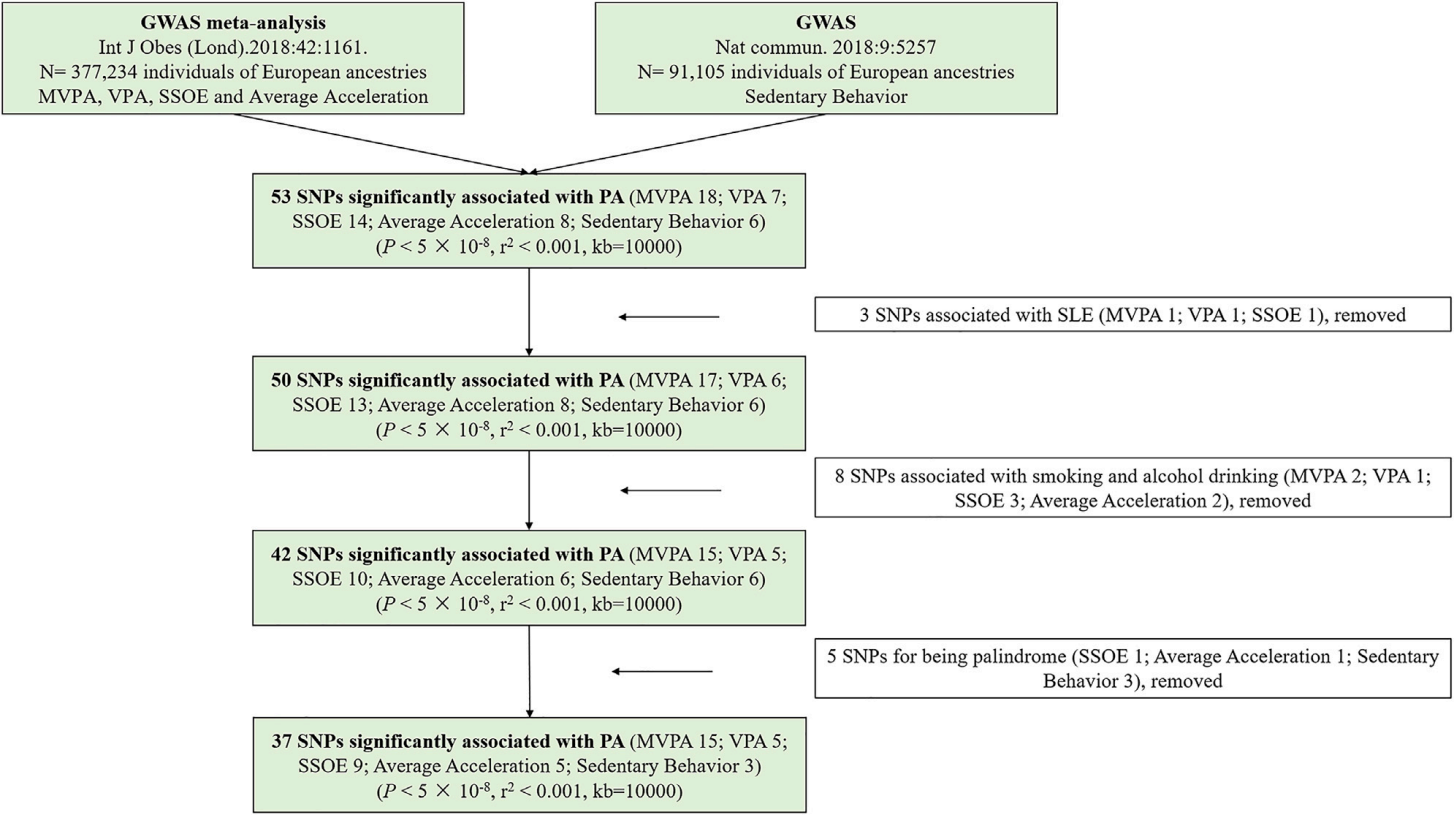
The flow chart of instrumental variables selection. Abbreviations: PA, physical activity; GWAS, genome-wide association study; SNPs, single nucleotide polymorphisms; MVPA, Moderate-to-vigorous physical activity; VPA, vigorous physical activity; SSOE, strenuous sports or other exercises.

In the present study, SNPs were defined as IVs (Lawlor et al., 2008). All requested SNPs conformed with the following conditions: 1. strongly correlated with exposure based on genome-wide significance; 2. having no linkage disequilibrium (LD) (pairwise *r* ^*2*^ < 0.001, window size = 10,000 kb); 3. without palindromic structures. According to the three mentioned assumptions and above conditions, a total of 37 SNPs were identified. To achieve powerful estimates, we used proxy SNPs with strong LD (*r* ^*2*^ > 0.8) to substitute for the selected SNPs on condition that the corresponding SNPs were unavailable in SLE GWAS. In the study, *F* statistic was calculated to quantify the strength of selected IVs (Burgess et al., 2019). We estimated the variance (R^**2**^) explained by every SNPs according to the equation of 2 × MAF × (1 − MAF) × β^**2**^ (Park et al., 2010 **)**. The R^**2**^ of each kind of PA was the sum of R^**2**^ of every SNP, which was powerfully related to it. Besides, the smallest effect detected by the sample size to provide 80% statistical power at an α level of 5% was computed at an online web tool (https://sb452.shinyapps.io/power/) (Burgess, 2014). Figure 2 displayed the flow chart of IVs selection.

### Statistical analysis

The inverse-variance weighted (IVW) method was conducted, as the primary method, to evaluate the causal association between PA and SLE (Burgess et al., 2019). We chose a fixed-effects model when the *p*-value, as the result of Cochran’s Q test, is >0.05, otherwise the random-effects model was applied (Higgins et al., 2003). The IVW method was perceived as the most dependable if the selected IVs did not have directional pleiotropy (*p*-value for MR-Egger intercept >0.05) (Holmes et al., 2017).

In sensitivity analyses, we chose MR-Egger method to evaluate the potential pleiotropy effects. The MR-Egger regression estimated the causal effect as the slope from the weighted regression of the IVs-outcome associations on the IVs-exposure associations, and the intercept term reflected the average pleiotropic effect (Bowden et al., 2015; Burgess and Thompson, 2017). Additionally, we also applied simple median, weighted median, and MR pleiotropy residual sum and outlier test (MR-PRESSO) methods to assess the presence of pleiotropy (Burgess et al., 2017; Verbanck et al., 2018). If more than 50% SNPs are effective IVs, the consistent estimates of causal effect would be provided by the weighted median (Burgess et al., 2017). Not only does MR-PRESSO detect pleiotropy, but also it can exclude the outlying SNPs and reassess the effect estimates (Verbanck et al., 2018). Meanwhile, leave-one-out analysis was performed to test the influence of outlying values. To remove the effect of other confounders, we also explored the pleiotropy of each selected SNPs at the GWAS threshold of statistical significance (*p*-value <5 × 10^−8^) by the PhenoScanner V2 database (http://www.phenoscanner.medschl.cam.ac.uk/) (Kamat et al., 2019).

All tests were two sided, and differences were considered as statistical significance (*p*-value <0.05), unless noted. All of the analyses were conducted using TwoSampleMR (V 0.5.6) (Hemani et al., 2018) and MR-PRESSO (V 1.0) (Verbanck et al., 2018) packages in R software (4.1.0).

## Results

Supplementary Table S2 provided detailed information on the selected SNPs. In the MR study, a total of 5 SNPs (SSOE: rs7627864; average acceleration: rs6775319; sedentary behavior: rs26579, rs25981, rs6870096) were excluded for palindrome. Ultimately, 37 SNPs (15 SNPs for MVPA, 5 SNPs for VPA, 9 SNPs for SSOE, 5 SNPs for average acceleration, 3 SNPs for sedentary behavior, all *p*-value <5 × 10^−8^, *r* ^*2*^ < 0.001) were selected as IVs, which included 3 proxy SNPs. The *F* statistics of the chosen SNPs ranged from 29.93 to 51.82, which were greater than the conventional threshold of 10, indicating that the selected SNPs could decrease the bias of causal analysis. Besides, the selected SNPs together explained ∼ 0.130%, ∼ 0.020%, ∼ 0.020%, ∼ 10.533%, and ∼ 0.110% of the variances for MVPA, VPA, SSOE, average acceleration, and sedentary behavior, respectively (Supplementary Table S3). Based on the sample size of the SLE GWAS meta-analysis, there was >80% power to detect associations of MVPA, VPA, SSOE, average acceleration, and sedentary behavior with the risk of SLE for an effect size (OR) of ∼ 0.834 (Supplementary Table S3). All IVs for genetically predicted PA have been certified and applied in recent other MR studies (Legge et al., 2020; Papadimitriou et al., 2020; Bahls et al., 2021; Julian et al., 2021). Additionally, all of them were irrelevant to smoking and alcohol drinking (Supplementary Table S4).

**FIGURE 3:**
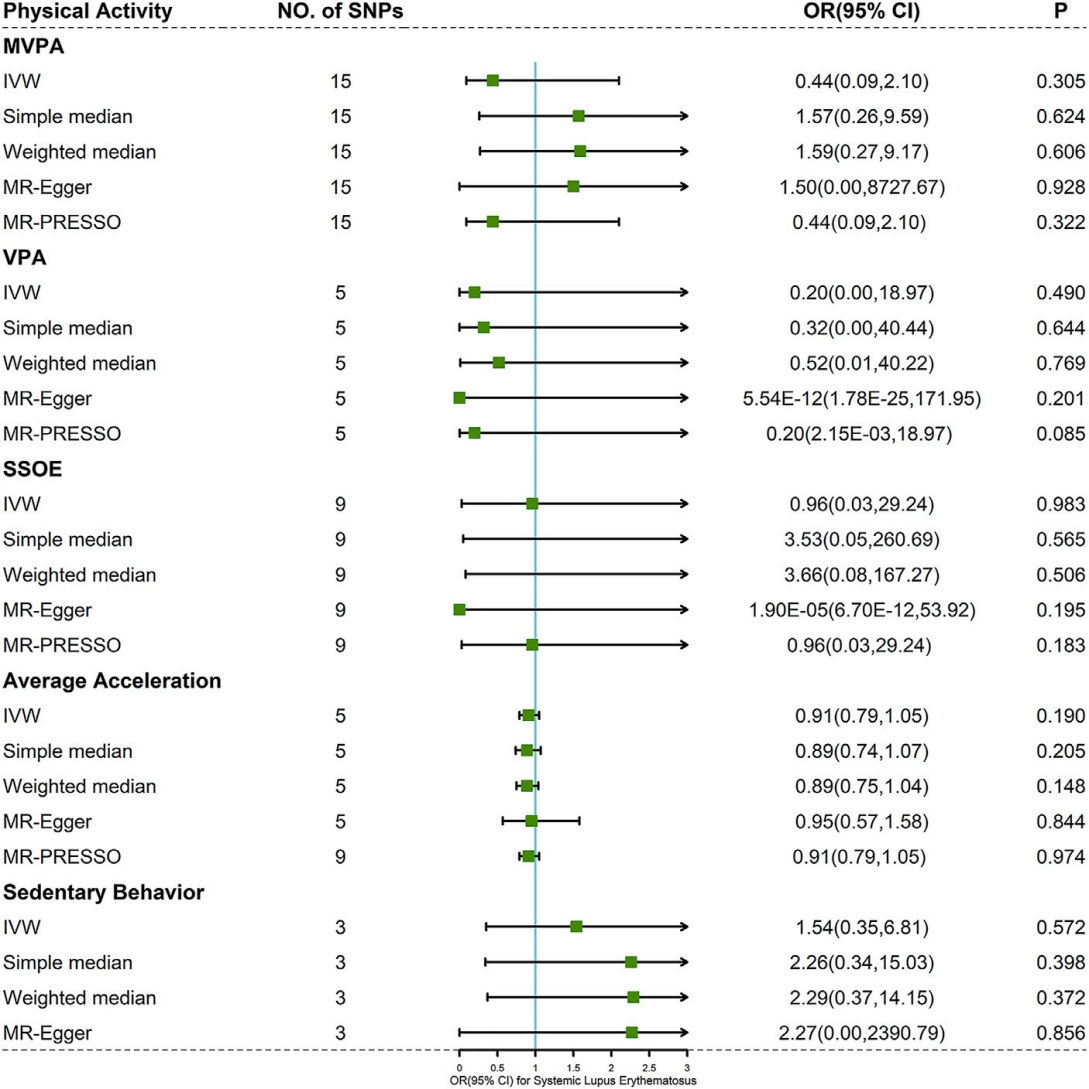
Forest plot of MR analysis for the causal association between PA and SLE. Abbreviations: MVPA, moderate-to-vigorous physical activity; VPA, vigorous physical activity; SSOE, strenuous sports or other exercises; OR, odds ratio; CI, confidence interval, IVW, inverse-variance weighted method; MR-Egger, Mendelian randomization-Egger; MR-PRESSO, Mendelian randomization pleiotropy residual sum and outlier test.

Cochran’s Q test indicated mild to moderate heterogeneity for MVPA, VPA, and SSOE (I^2^ = 47.389, *p*-value = 0.022 for MVPA; I^2^ = 56.937, *p*-value = 0.054 for VPA; I^2^ = 32.609, *p*-value = 0.157 for SSOE), average acceleration and sedentary behavior having no significant heterogeneity (I^2^ = 0.000, *p*-value = 0.971 for average acceleration; I^2^ = 0.000, *p*-value = 0.598 for sedentary behavior) (Supplementary Table S5). Based on the result of IVW method, there was little evidence supporting the causality between PA and risk of SLE (MVPA: OR 0.44, 95% CI 0.09–2.10, *p*-value = 0.305; VPA: OR 0.20, 95% CI 0.00–18.97, *p*-value = 0.490; SSOE: OR 0.96, 95% CI 0.03–29.24, *p*-value = 0.983; average acceleration: OR 0.91, 95% CI 0.79–1.05, *p*-value = 0.190; sedentary behavior: OR 1.54, 95% CI 0.35–6.81, *p*-value = 0.572) (Figure 3 and Supplementary Figure S1). The consequences of simple median and weighted median were similar to that of the IVW method. Meanwhile, the MR-Egger regression did not reveal horizontal pleiotropy (*P*_intercept_ = 0.783 for MVPA, *P*_intercept_ = 0.220 for VPA, *P*_intercept_ = 0.187 for SSOE, *P*_intercept_ = 0.890 for average acceleration, and *P*_intercept_ = 0.929 for sedentary behavior) (Supplementary Table S5). Similarly, the pleiotropy was not detected by MR-PRESSO **(** Figure 3) and PhenoScanner V2 database. Forest plots, scatter plots, funnel plots, and MR leave-one-out plots for PA are presented in Supplementary Figures S1–S4.

## Discussion

In this study, we found that there was no evidence to support the causal relationship between genetically predicted PA (MVPA, VPA, SSOE, average acceleration, and sedentary behavior) and risk of SLE among European populations by two-sample MR analysis. Sensitivity analyses also indicated that results were robust in general.

Previous observational study has demonstrated that SLE patients with longer sedentary behavior presented increased disease activity and reduced scores of physical component of QoL (Margiotta et al., 2018). Another randomized controlled trial also indicated that SLE patients had lower exercise capacity and less frequent exercise than healthy controls (Eriksson et al., 2012). Therefore, they advocated that it was necessary for SLE patients to enhance the awareness of increasing PA and reducing sedentary behavior to control the disease activity. Even though the etiology of SLE is unclear, the depletion of Treg cells and activation of macrophages played an important role in the pathogenesis (Yang et al., 2009; Al Gadban et al., 2015). Previous studies found exercise was able to decrease the antigen-presenting function by downregulating TLR expression in macrophages and induce the increase of Treg cells (Sharif et al., 2018).

However, several clinical studies reported that there was an insignificant difference between exercise and control groups in SLE-related damage and disease activity (Abrahao et al., 2016; Bostrom et al., 2016). Similar results were reported in a meta-analysis consisting of 11 randomized controlled trials (O'Dwyer et al., 2017). In addition, our MR study also indicated that there was little evidence for causality between PA and the risk of SLE. These discrepant findings might result from reverse causality bias. For example, SLE patients presented less frequent exercise and longer sedentariness due to higher disease activity, joint pain or fatigue.

Although PA could not reduce disease activity, organ damage, and risk of SLE, it may have other positive effects. Previous research indicated that PA could improve fatigue, psychological function, quality of life, etc. (Fangtham et al., 2019). Increased risk of cardiovascular diseases (CVDs) was also associated with immunological dysregulation and inflammation due to SLE (Avina-Zubieta et al., 2017). However, MVPA could decrease the cardiovascular risk of patients with SLE (Legge et al., 2020). What is more, the European League Against Rheumatism (EULAR) administered PA as adjuvant therapy in SLE patients with increased risk of CVDs, particularly (Bertsias et al., 2008).

Among the selected SNPs, some were suggested to be associated with immunity. For example, PAX4 (rs2988004) was B-cell transcription factor genes, playing an essential role in controlling the identity and function of B cells throughout B lymphopoiesis (Cobaleda et al., 2007). ACYP2 (rs1974771) promoted phosphorylation and activity of STAT3, which played critical functions in the differentiation of follicular helper T cell (Tripathi and Lahesmaa, 2014). FOXO1 (rs2764261) enhanced differentiation, proliferation, immunoglobulin gene rearrangement, and class switching in B cells (Cabrera-Ortega et al., 2017). In CD4^+^ T cells of MRL/lpr mice, CTBP2 (rs3781411) suppressed various genes, including IL-2 (Katsuyama et al., 2018). Besides, SKI (rs61776614) inhibited pathogenic Th17 cell response and ameliorated experimental autoimmune encephalomyelitis (Li et al., 2021).

As far as we know, this study is the first MR study to analyze whether PA is causally concerned with risk of SLE based on open GWAS databases. Furthermore, to decrease the population bias, we selected European individuals for this two-sample MR study. Finally, to avoid information bias, we collected five types of PA, which were evaluated by subjective and objective assessments. MVPA, VPA, and SSOE were measured by a touchscreen questionnaire, and average acceleration and sedentary behavior were objectively assessed by a wrist-worn accelerometer.

The present MR study also had several limitations. First, our study was based on openly available genetic data, and we could not perform stratified analyses or analyses adjusted for other covariates. Second, the selected instrumental SNPs as IVs explained relatively limited proportion of variance in PA, ranging from 0.020% to 10.533%. This may lead to low statistical power to detect weak associations. In the end, our data source was obtained from individuals of European ancestry, which did not necessarily generalize our findings to other populations outside Europe.

In conclusion, our MR study indicates that genetically predicted PA is not causally associated with risk of SLE among European individuals. More researches are required to explore the causal relationship between PA and SLE.

## Data Availability

The original contributions presented in the study are included in the article/Supplementary Material, Further inquiries can be directed to the corresponding authors.
