# Supplementary material for: Physical Activity and Systemic Lupus Erythematosus Among European Populations: A Two-Sample Mendelian Randomization Study
Source: Front Genet. 2022 Feb 8;12:784922. doi: 10.3389/fgene.2021.784922 (PMC8861300; doi:10.3389/fgene.2021.784922)
Supplement: Supplementary file 2 [file DataSheet1.docx]

SUPPLEMENTAL MATERIALS

Supplemental Table S1. Descriptive details about data sources of physical activity (PA), smoking, alcohol drinking and SLE

Supplemental Table S2. Summary statistics for the genetic variants associated with physical activity (PA) and systemic lupus erythematosus (SLE)

Supplemental Table S3. Variances explained by the selected instruments and power calculations for the Mendelian randomization (MR) analysis among European populations

Supplemental Table S4. The Associations between SNPs and smoking, alcohol drinking

Supplemental Table S5. The result of sensitivity analyses by Cochran’s Q Test and MR-Egger Test for physical activity (PA)

Supplemental Figure S1. Forest plots of SNPs associated with physical activity (PA) and systemic lupus erythematosus (SLE)

Supplemental Figure S2. Scatter plots of SNPs associated with physical activity (PA) and systemic lupus erythematosus (SLE)

Supplemental Figure S3. Funnel plots of SNPs associated with physical activity (PA) and systemic lupus erythematosus (SLE)

Supplemental Figure S4. Leave-one-out plots of SNPs associated with physical activity (PA) and systemic lupus erythematosus (SLE)

Supplemental Table S1. Descriptive details about data sources of physical activity (PA), smoking, alcohol drinking and SLE

| Exposure or outcome | First author (year) | Participants (Sample size) | Web source |
| --- | --- | --- | --- |
| PA |  |  |  |
| MVPA | Klimentidis YC (2018) | 377,234 individuals of European ancestries | Int J Obes (Lond). 2018:42:1161. |
| VPA | Klimentidis YC (2018) | 261,055 individuals of European ancestries |  |
| SSOE | Klimentidis YC (2018) | 350,492 individuals of European ancestries |  |
| Average Acceleration | Klimentidis YC (2018) | 91,084 individuals of European ancestries |  |
| Sedentary Behavior | Doherty A (2018) | 91,105 individuals of European ancestries | Nat commun. 2018:9:5257. |
| SLE | Bentham J (2015) | 4,036 SLE cases and 6,959 controls of European ancestries | Nat Genet. 2015:47:1457. |
| Smoking  (Cigarettes per Day) | Liu M (2019) | 337,334 individuals of European ancestries | Nat Genet. 2019:51:237. |
| Alcohol drinking  (Alcoholic drinks per week) | Liu M (2019) | 941,280 individuals of European ancestries |  |

Abbreviations: MVPA: Moderate-to-vigorous physical activity; VPA: vigorous physical activity; SSOE: strenuous sports or other exercises; SLE: systemic lupus erythematosus

Supplemental Table S2. Summary statistics for the genetic variants associated with physical activity (PA) and systemic lupus erythematosus (SLE)

| Physical Activity | SNP | Gene | chr | Pos | EA | OA | EAF | Physical Activity | | | | Number of individuals involved | SLE* | | |
| --- | --- | --- | --- | --- | --- | --- | --- | --- | --- | --- | --- | --- | --- | --- | --- |
|  |  |  |  |  |  |  |  | Beta | SE | *P*-value | *F* statistic |  | Beta | SE | *P*-value |
| MVPA | rs1972763 | *C4orf45* | 4 | 159868813 | T | C | 0.658 | -0.0128 | 0.0023 | 3.30E-08 | 30.53 | 377,234 | 0.0101 | 0.0359 | 0.779 |
|  | rs1921981 | *-* | 21 | 42422547 | A | G | 0.326 | -0.0130 | 0.0024 | 3.80E-08 | 30.22 | 377,234 | -0.0408 | 0.0352 | 0.246 |
|  | rs7804463 | *EXOC4* | 7 | 133447651 | C | T | 0.470 | -0.0150 | 0.0022 | 1.20E-11 | 45.99 | 377,234 | -0.0408 | 0.0272 | 0.133 |
|  | rs877483 | *CACNA1D* | 3 | 53846741 | C | T | 0.567 | -0.0122 | 0.0022 | 4.00E-08 | 30.13 | 377,234 | -0.0100 | 0.0194 | 0.608 |
|  | rs921915 | *AC020743.3* | 7 | 50228581 | C | T | 0.588 | 0.0139 | 0.0022 | 5.70E-10 | 38.44 | 377,234 | -0.0770 | 0.0282 | 0.006 |
|  | rs12912808 | *RP11-255M2.3* | 15 | 95292223 | T | C | 0.149 | -0.0175 | 0.0031 | 1.70E-08 | 31.85 | 377,234 | 0.0583 | 0.0404 | 0.150 |
|  | rs1043595 | *CALU* | 7 | 128410012 | A | G | 0.283 | -0.0144 | 0.0025 | 4.30E-09 | 34.48 | 377,234 | -0.0202 | 0.0369 | 0.584 |
|  | rs2988004 | *PAX5* | 9 | 37044388 | G | T | 0.442 | 0.0132 | 0.0022 | 4.10E-09 | 34.58 | 377,234 | -0.0513 | 0.0293 | 0.080 |
|  | rs1974771 | *ACYP2* | 2 | 54278543 | A | G | 0.100 | 0.0213 | 0.0037 | 6.60E-09 | 33.65 | 377,234 | 0.0100 | 0.0392 | 0.800 |
|  | rs10145335 | *LOC105370655* | 14 | 98547748 | A | G | 0.251 | 0.0141 | 0.0025 | 2.70E-08 | 30.88 | 377,234 | -0.1054 | 0.0358 | 0.003 |
|  | rs2114286 | *RP11-944L7.4* | 3 | 41194283 | G | A | 0.534 | 0.0122 | 0.0022 | 3.30E-08 | 30.50 | 377,234 | 0.0101 | 0.0360 | 0.780 |
|  | rs429358 | *APOE* | 19 | 45411941 | C | T | 0.154 | 0.0220 | 0.0031 | 6.10E-13 | 51.82 | 377,234 | 0.0100 | 0.0411 | 0.809 |
|  | rs1186721 | *DPY19L1* | 7 | 34974602 | A | G | 0.316 | 0.0130 | 0.0024 | 4.40E-08 | 29.98 | 377,234 | -0.0513 | 0.0327 | 0.117 |
|  | rs77742115 | *CTD-2533K21.1* | 5 | 18330424 | C | T | 0.138 | 0.0183 | 0.0032 | 9.60E-09 | 32.92 | 377,234 | -0.0202 | 0.0500 | 0.686 |
|  | rs4886868 | *DNM1P33* | 15 | 74353561 | G | T | 0.586 | 0.0125 | 0.0023 | 3.50E-08 | 30.40 | 377,234 | 0.0202 | 0.0376 | 0.591 |
| VPA | rs2764261 | *FOXO3* | 6 | 108927842 | G | A | 0.626 | -0.0091 | 0.0014 | 2.00E-11 | 45.00 | 261,055 | -0.0296 | 0.0291 | 0.310 |
|  | rs13243553 | *EXOC4* | 7 | 133506955 | A | G | 0.392 | -0.0087 | 0.0013 | 9.00E-11 | 42.02 | 261,055 | -0.0305 | 0.0331 | 0.357 |
|  | rs6667222 | *HAX1* | 1 | 154253661 | C | A | 0.252 | -0.0087 | 0.0015 | 8.70E-09 | 33.10 | 261,055 | 0.0100 | 0.0268 | 0.711 |
|  | rs328902 | *DPY19 L1* | 7 | 35020843 | T | C | 0.315 | 0.0088 | 0.0014 | 5.50E-10 | 38.48 | 261,055 | -0.0513 | 0.0328 | 0.118 |
|  | rs3781411 | *CTBP2* | 10 | 126715436 | T | C | 0.124 | -0.0126 | 0.0020 | 3.00E-10 | 39.67 | 261,055 | 0.1044 | 0.0428 | 0.015 |
| SSOE | rs75930676 | *SIPA1 L1* | 14 | 71826547 | C | T | 0.051 | 0.0158 | 0.0026 | 2.00E-09 | 36.01 | 350,492 | -0.0834 | 0.0721 | 0.247 |
|  | rs166840 | *AKAP10* | 17 | 19799698 | A | G | 0.412 | -0.0076 | 0.0012 | 3.10E-11 | 44.13 | 350,492 | 0.0100 | 0.0281 | 0.724 |
|  | rs159544 | *CTC-436P18.1* | 5 | 60489247 | G | A | 0.395 | 0.0070 | 0.0012 | 1.30E-09 | 36.76 | 350,492 | 0.0488 | 0.0280 | 0.081 |
|  | rs288070 | *MAP2* | 2 | 210408477 | A | G | 0.098 | 0.0106 | 0.0019 | 1.90E-08 | 31.57 | 350,492 | -0.0834 | 0.0485 | 0.086 |
|  | rs2994326 | *SDCCAG8* | 1 | 243651026 | C | T | 0.813 | 0.0080 | 0.0015 | 4.50E-08 | 29.93 | 350,492 | 0.0101 | 0.0405 | 0.804 |
|  | rs896302 | *RP11-436D23.1* | 6 | 98806496 | T | C | 0.713 | -0.0070 | 0.0012 | 1.70E-08 | 31.76 | 350,492 | -0.0100 | 0.0371 | 0.789 |
|  | rs4411372 | *STK24* | 13 | 99130423 | C | T | 0.280 | 0.0070 | 0.0013 | 2.00E-08 | 31.46 | 350,492 | -0.0408 | 0.0321 | 0.203 |
|  | rs1200154 | *ATP1B1* | 1 | 169093101 | A | G | 0.593 | 0.0063 | 0.0011 | 3.90E-08 | 30.21 | 350,492 | 0.0101 | 0.0199 | 0.613 |
|  | rs111901094 | *GATAD2A* | 19 | 19642795 | T | G | 0.182 | -0.0088 | 0.0015 | 3.00E-09 | 35.16 | 350,492 | -0.0726 | 0.0468 | 0.121 |
| Average Acceleration | rs148193266 | *RP11-681H10.1* | 11 | 104528681 | C | A | 0.043 | 0.5104 | 0.0922 | 3.10E-08 | 30.67 | 91,084 | -0.0202 | 0.0765 | 0.792 |
|  | rs12522261 | *LINC01470* | 5 | 152054825 | A | G | 0.343 | -0.2105 | 0.0383 | 3.90E-08 | 30.21 | 91,084 | -0.0101 | 0.0584 | 0.863 |
|  | rs34517439 | *DNAJB4* | 1 | 78450517 | A | C | 0.121 | -0.3079 | 0.0562 | 4.40E-08 | 29.97 | 91,084 | 0.0392 | 0.0539 | 0.467 |
|  | rs11012732 | *MLLT10* | 10 | 21859585 | G | A | 0.332 | -0.2250 | 0.0386 | 5.40E-09 | 34.04 | 91,084 | 0.0296 | 0.0344 | 0.391 |
|  | rs9293503 | *LINC00461* | 5 | 87948962 | C | T | 0.112 | -0.3290 | 0.0587 | 2.10E-08 | 31.42 | 91,084 | 0.0392 | 0.0418 | 0.348 |
| Sedentary Behavior | rs61776614 | *SKI* | 1 | 2166406 | C | T | 0.925 | 0.0500 | 0.0090 | 4.70E-08 | 30.86 | 91,105 | 0.0408 | 0.0696 | 0.558 |
|  | rs1858242 | *LOC105377146* | 3 | 68527135 | A | G | 0.259 | 0.0310 | 0.0050 | 3.10E-09 | 38.44 | 91,105 | -0.0202 | 0.0409 | 0.621 |
|  | rs34858520 | *CALN1* | 7 | 71723883 | A | G | 0.558 | 0.0280 | 0.0050 | 4.20E-09 | 31.36 | 91,105 | 0.0305 | 0.0349 | 0.383 |

Note: According to Bonferroni correction, only P values lower than 0.00068 (0.05/37 SNPs/2 phenotypes) were considered statistically significant. F statistic = Beta² / SE² (Georgakis et al., 2019). *Included SNPs: rs1972763 (proxy: rs1834682); rs111901094 (proxy: rs56397647); rs11012732 (proxy: rs61850044).

Abbreviations: SNP: single-nucleotide polymorphisms; Chr: chromosome; Pos: position; EA: effect allele; OA: other allele, EAF: effect allele frequency; SE: standard error.

Supplemental Table S3. Variances explained by the selected instruments and power calculations for the Mendelian randomization (MR) analysis among European populations

| Physical Activity | Variance explained (R^2^, %) | SLE GWAS Sample size | Ratio of cases to control | Odds ratio estimated for α^1^ |
| --- | --- | --- | --- | --- |
| MVPA | 0.130 | 10,995 | 1:1.724 | 0.215 |
| VPA | 0.020 |  |  | 0.020 |
| SSOE | 0.020 |  |  | 0.020 |
| Average Acceleration | 10.533 |  |  | 0.843 |
| Sedentary Behavior | 0.110 |  |  | 0.188 |

Note: ^1^ Odds ratio estimated for α, the smallest effect detected by the sample size to provide 80% statistical power at an alpha level of 5%.

Supplemental Table S4. The Associations between SNPs and smoking, alcohol drinking

| Physical Activity | SNP | Alcohol drinking | | |  | Smoking | | |
| --- | --- | --- | --- | --- | --- | --- | --- | --- |
|  |  | Beta | SE | *P*-value |  | Beta | SE | *P*-value |
| MVPA | rs1972763 | -0.00162 | 0.00204 | 0.426 |  | 0.00181 | 0.00887 | 0.838 |
|  | rs1921981 | 0.00310 | 0.00206 | 0.133 |  | 0.00207 | 0.00589 | 0.725 |
|  | rs7804463 | -0.00111 | 0.00193 | 0.565 |  | -0.01247 | 0.00552 | 0.024 |
|  | rs877483 | -0.00232 | 0.00195 | 0.234 |  | -0.00183 | 0.00556 | 0.743 |
|  | rs921915 | 0.00022 | 0.00196 | 0.912 |  | 0.00183 | 0.00561 | 0.744 |
|  | rs12912808 | 0.00179 | 0.00269 | 0.505 |  | 0.00521 | 0.00763 | 0.494 |
|  | rs1043595 | -0.00279 | 0.00216 | 0.195 |  | 0.01080 | 0.00616 | 0.080 |
|  | rs2988004 | 0.00159 | 0.00195 | 0.413 |  | -0.00169 | 0.00556 | 0.761 |
|  | rs1974771 | 0.00231 | 0.00315 | 0.463 |  | -0.00593 | 0.00582 | 0.308 |
|  | rs10145335 | -0.00101 | 0.00224 | 0.652 |  | 0.01455 | 0.00638 | 0.023 |
|  | rs2114286 | -0.00131 | 0.00193 | 0.497 |  | -0.00704 | 0.00552 | 0.202 |
|  | rs429358 | -0.00312 | 0.00269 | 0.247 |  | -0.02010 | 0.00776 | 0.010 |
|  | rs1186721 | -0.00080 | 0.00209 | 0.703 |  | -0.01522 | 0.00597 | 0.011 |
|  | rs77742115 | -0.00464 | 0.00276 | 0.092 |  | -0.00614 | 0.00777 | 0.430 |
|  | rs4886868 | 0.00118 | 0.00195 | 0.545 |  | -0.00175 | 0.00562 | 0.756 |
| VPA | rs2764261 | 0.00114 | 0.00199 | 0.566 |  | 0.00582 | 0.00571 | 0.308 |
|  | rs13243553 | 0.00024 | 0.00197 | 0.901 |  | -0.00766 | 0.00563 | 0.174 |
|  | rs6667222 | 0.00154 | 0.00223 | 0.489 |  | 0.00677 | 0.00637 | 0.287 |
|  | rs328902 | -0.00136 | 0.00208 | 0.516 |  | -0.01458 | 0.00596 | 0.015 |
|  | rs3781411 | -0.00027 | 0.00289 | 0.925 |  | 0.01137 | 0.00819 | 0.165 |
| SSOE | rs75930676 | 0.00524 | 0.00449 | 0.243 |  | 0.01722 | 0.01305 | 0.187 |
|  | rs166840 | -0.00276 | 0.00197 | 0.160 |  | -0.00690 | 0.00561 | 0.219 |
|  | rs159544 | -0.00144 | 0.00198 | 0.467 |  | 0.00198 | 0.00565 | 0.726 |
|  | rs288070 | 0.00428 | 0.00317 | 0.178 |  | -0.01078 | 0.00898 | 0.231 |
|  | rs2994326 | 0.00278 | 0.00249 | 0.265 |  | -0.01102 | 0.00713 | 0.122 |
|  | rs896302 | -0.00526 | 0.00213 | 0.013 |  | 0.00667 | 0.00607 | 0.271 |
|  | rs4411372 | 0.00340 | 0.00215 | 0.114 |  | -0.01380 | 0.00624 | 0.027 |
|  | rs1200154 | -0.00003 | 0.00196 | 0.988 |  | -0.01781 | 0.00561 | 0.001 |
|  | rs111901094 | 0.00528 | 0.00302 | 0.081 |  | 0.01521 | 0.00860 | 0.077 |
| Average Acceleration | rs148193266 | 0.00266 | 0.00476 | 0.575 |  | -0.00047 | 0.01368 | 0.972 |
|  | rs12522261 | 0.00236 | 0.00203 | 0.246 |  | -0.01086 | 0.00581 | 0.061 |
|  | rs34517439 | -0.00290 | 0.00298 | 0.331 |  | 0.02534 | 0.00844 | 0.003 |
|  | rs11012732 | -0.00085 | 0.00205 | 0.678 |  | -0.00505 | 0.00585 | 0.388 |
|  | rs9293503 | 0.00704 | 0.00319 | 0.027 |  | -0.00609 | 0.00924 | 0.510 |
| Sedentary Behavior | rs61776614 | 0.00230 | 0.00364 | 0.529 |  | -0.00890 | 0.01022 | 0.384 |
|  | rs1858242 | -0.00099 | 0.00221 | 0.655 |  | -0.00579 | 0.00631 | 0.359 |
|  | rs34858520 | -0.00403 | 0.00194 | 0.038 |  | -0.00925 | 0.00555 | 0.095 |

Note: According to Bonferroni correction, only P values lower than 0.00068 (0.05/37 SNPs/2 phenotypes) were considered statistically significant.

Supplemental Table S5. Results for MR-Egger (intercept), Cochran’s Q test of physical activity (PA) for systemic lupus erythematosus (SLE).

| Physical Activity | MR-Egger Test | | |  | Cochran’s Q Test | | | |
| --- | --- | --- | --- | --- | --- | --- | --- | --- |
|  | Intercept | SE | *P*-value |  | Q | Q_df | I^2^ | *P*-value |
| MVPA | -0.018 | 0.064 | 0.783 |  | 26.611 | 14 | 47.389 | 0.022 |
| VPA | 0.229 | 0.148 | 0.220 |  | 9.289 | 4 | 56.937 | 0.054 |
| SSOE | 0.084 | 0.058 | 0.187 |  | 11.871 | 8 | 32.609 | 0.157 |
| Average Acceleration | -0.012 | 0.077 | 0.890 |  | 0.528 | 4 | 0.000 | 0.971 |
| Sedentary Behavior | -0.013 | 0.116 | 0.929 |  | 1.029 | 2 | 0.000 | 0.598 |

Note: I^2^ was applied to estimate heterogeneity by the equation of (Q-Q_df) × 100%/Q. And negative values of I^2^ are put equal to zero(Higgins et al., 2003). Abbreviations: MR-Egger: Mendelian randomization-Egger.

Supplemental Figure S1. Forest plots of SNPs associated with physical activity (PA) and systemic lupus erythematosus (SLE).


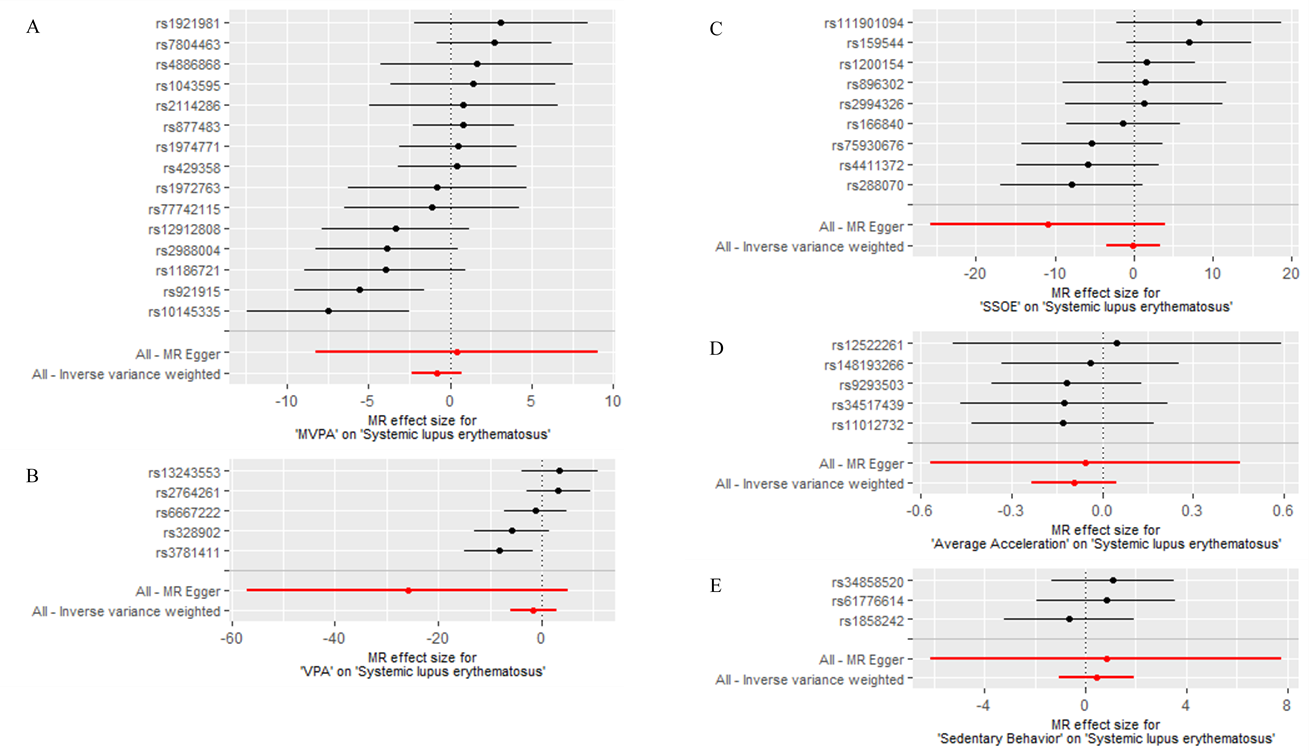


Note: Each row (black lines) represents the result after the removal of the corresponding SNP, and the overall effect is shown at the bottom (red line). The examined physical activity included (A) MVPA, (B) VPA, (C) SSOE, (D) average acceleration and (E) sedentary behavior.


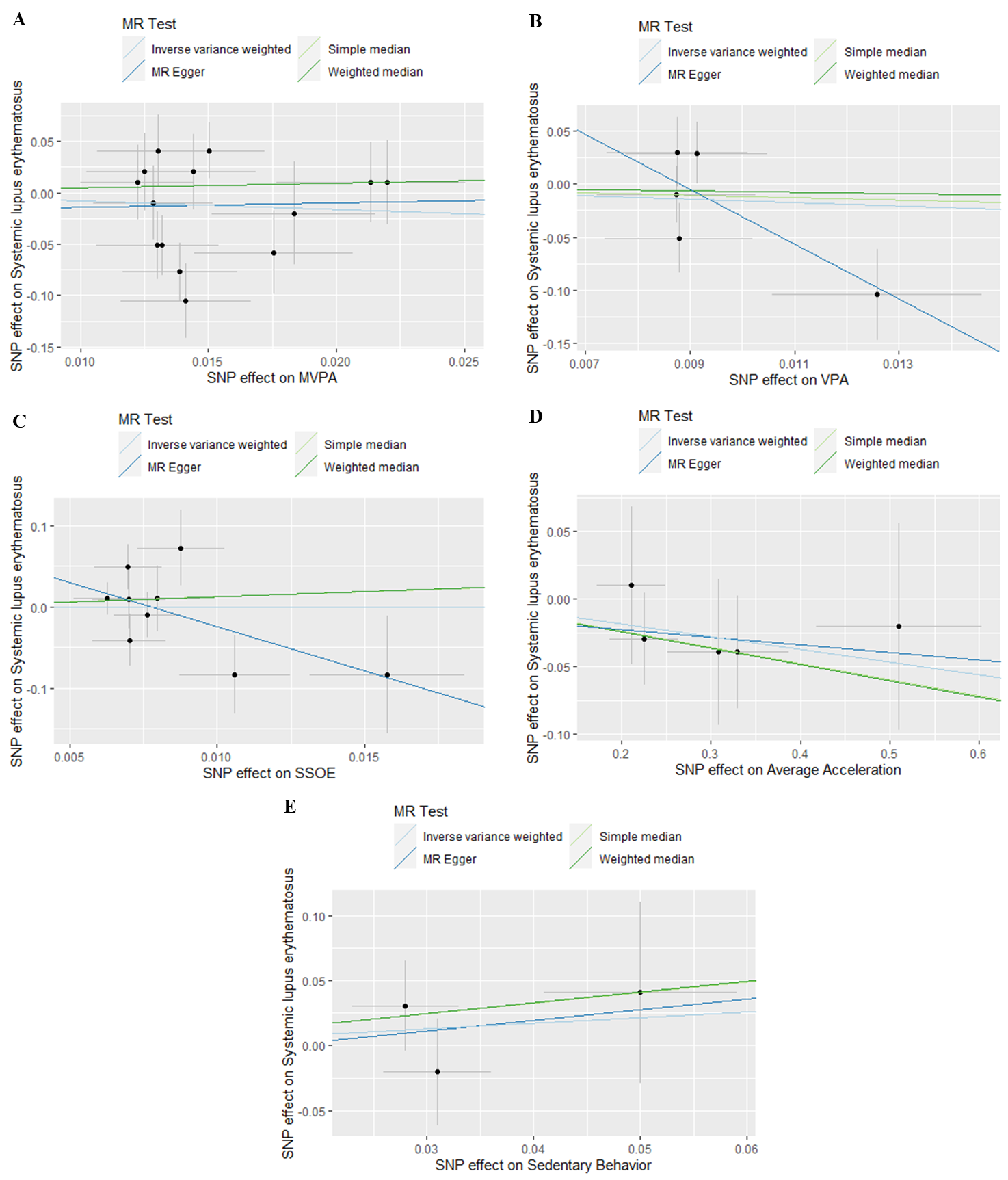
Supplemental Figure S2. Scatter plots of SNPs associated with a physical activity (PA) and systemic lupus erythematosus (SLE)

Note: According to the catter plots, the risk of RA was increased as adipokines levels became higher. The examined physical activity included (A) MVPA, (B) VPA, (C) SSOE, (D) average acceleration and (E) sedentary behavior.

Supplemental Figure S3. Funnel plots of SNPs associated with physical activity (PA) and systemic lupus erythematosus (SLE).


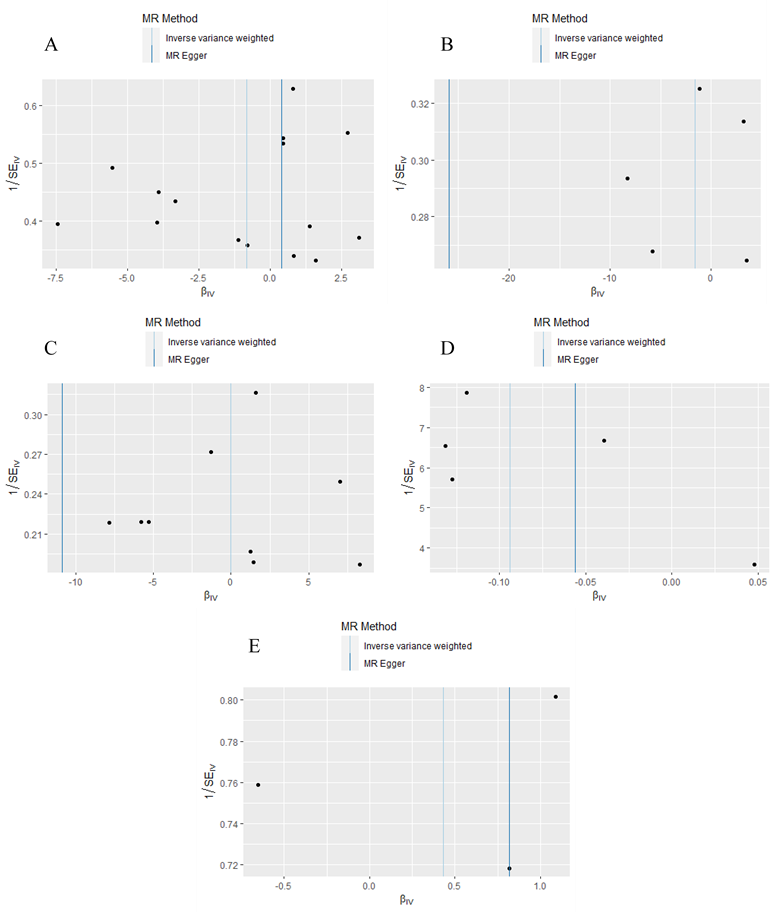


Note: The funnel plot showed that there was basically symmetry. The examined physical activity included (A) MVPA, (B) VPA, (C) SSOE, (D) average acceleration and (E) sedentary behavior.

Supplemental Figure S4. Leave-one-out plots of SNPs associated with physical activity (PA) and systemic lupus erythematosus (SLE).


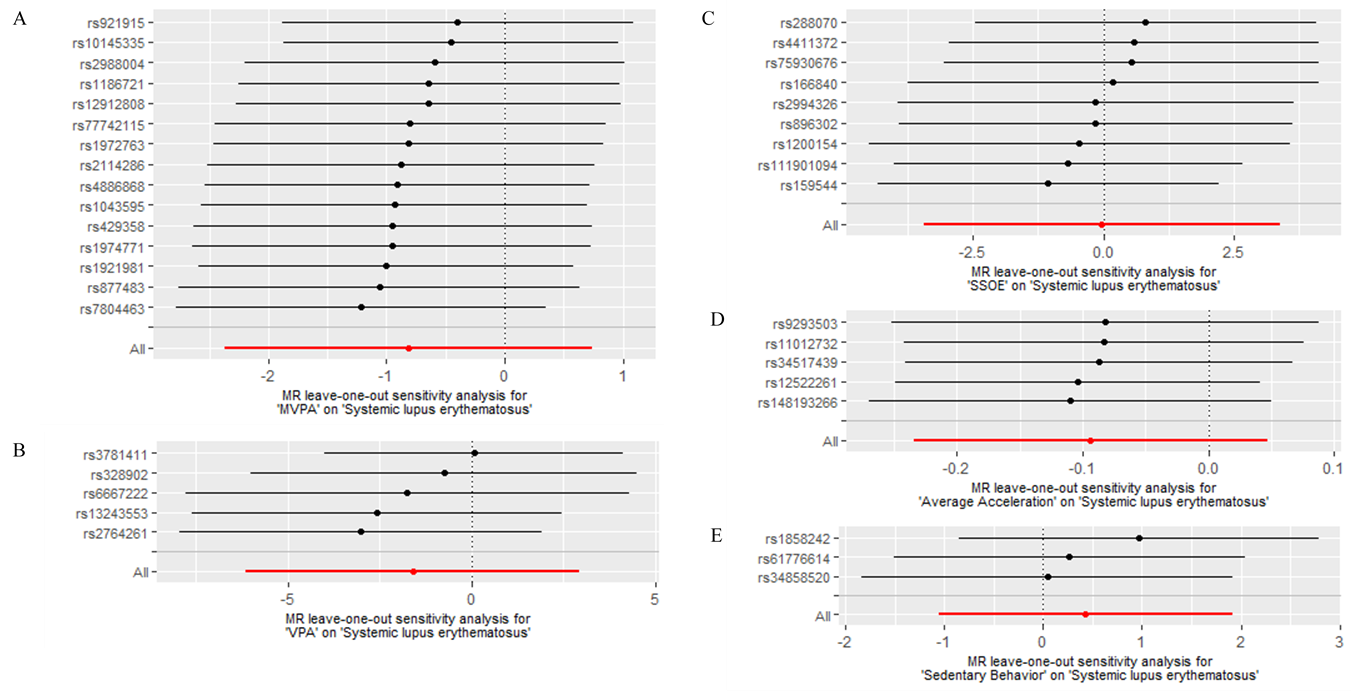


Note: Each row (black lines) represents the result after the removal of the corresponding SNP, and the overall effect is shown at the bottom (red line). The examined physical activity included (A) MVPA, (B) VPA, (C) SSOE, (D) average acceleration and (E) sedentary behavior.

References

Georgakis, M.K., Malik, R., Bjorkbacka, H., Pana, T.A., Demissie, S., Ayers, C., et al. (2019). Circulating Monocyte Chemoattractant Protein-1 and Risk of Stroke: Meta-Analysis of Population-Based Studies Involving 17 180 Individuals. *Circ Res* 125(8), 773-782. doi: 10.1161/CIRCRESAHA.119.315380.

Higgins, J.P., Thompson, S.G., Deeks, J.J., and Altman, D.G. (2003). Measuring inconsistency in meta-analyses. *BMJ* 327(7414), 557-560. doi: 10.1136/bmj.327.7414.557.
